# Silver nanoparticle@carbon quantum dot composite as an antibacterial agent†

**DOI:** 10.1039/d2ra00561a

**Published:** 2022-03-28

**Authors:** Tianyu Liu, Qianyue Pang, Kang Mai, Xiaoting He, Li Xu, Feiyan Zhou, Yi Liu

**Affiliations:** School of Pharmacy, Guangdong Pharmaceutical University Guangzhou 510006 China liuyi915@126.com; School of Pharmaceutical and Chemical Engineering, Guangdong Pharmaceutical University Zhongshan 528458 China; Zhongshan Carefor Daily Necessities Ltd Zhongshan 528000 China; Guangzhou Baiyunshan Weiyi Industrial Co., Ltd Guangzhou 510000 China

## Abstract

A AgNPs@S,N-CQDs composite was synthesized by a one-step approach. It possessed AgNPs naturally surrounded by S,N-CQDs, and the size of the particles was found to be uniform and stable *via* a series of characterization methods. The antibacterial properties of the composite material were studied, and it had good antibacterial properties against *S. aureus*, *E. coli*, MRSA and *C. albicans*. The minimum inhibitory concentrations were 63 μg mL^−1^ against *S. aureus* and MRSA and 32 μg mL^−1^ against *E. coli* and *C. albicans*. In addition, the AgNPs@S,N-CQDs composite had an antibacterial effect *via* the generation of ROS, which was verified using the DCFH-DA kit. Finally, HepG2 cells were used to study its biocompatibility. The antibacterial properties and biocompatibility results show that the AgNPs@S,N-CQDs composite material can serve as a promising antibacterial agent.

## Abstract 2

Synthesis of AgNPs@S,N-CQDs active against bacteria.

## Graphical abstract

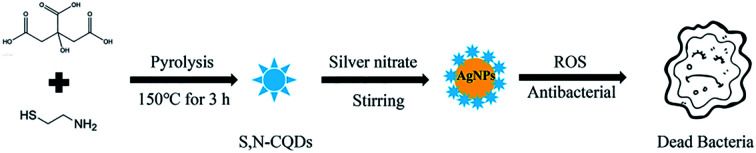

## 1. Introduction

Bacterial infections are considered a major threat to public health.^1^ The most effective treatment for pathogenic bacteria in the medical system is the use of antibiotics.^2^ The limitation of antibiotics is that some antibiotics only have antibacterial effects against a certain type of pathogenic bacteria, and do not have broad-spectrum antibacterial properties.^3^ This also leads to a serious problem wherein certain bacteria are prone to becoming resistant to antibiotics.^4^ Due to the rapid emergence of antibiotic-resistant strains, there is an urgent need to find alternatives to antibiotics.^5^

Nanoparticles have shown great potential in solving the problem of bacterial multidrug resistance and are regarded as viable alternatives to antibiotics.^6^ Silver nanoparticles (AgNPs) show special prospects and can be used as a medical antibacterial agent against both Gram-positive and Gram-negative bacteria.^7^ AgNPs exhibit a bactericidal effect by destroying bacterial cell membranes and inducing the release of reactive oxygen species (ROS) to form free radicals with powerful bactericidal ability.^8^ Hence, numerous studies have demonstrated that the synergistic effect of AgNPs with other materials can enhance antibacterial activity, while also efficiently decreasing the size and preventing the aggregation of AgNPs.^9^ Finding suitable surface passivation or support materials for silver nanoparticles can retain their antibacterial activity while having better stability, which can solve the above problem well.^10^ Kadian *et al.* reported a simple one-step synthesis of a silver nanoparticle nanocomposite modified by sulfur-doped graphene quantum dots (S-GQDs). The results showed that the minimum inhibitory concentration (MIC) values of the Ag@S-GQDs nanocomposite prepared by this method were 70 and 35 μg mL^−1^, which were sufficient to hinder the growth of *P. aeruginosa* and *S. aureus*, respectively.^11^

Currently, the preparation of materials with high antibacterial activity has broad prospective application. CQDs are a new type of metal-free fluorescent nanoparticle,^12^ and have drawn wide attention from scholars due to their simple synthesis, low toxicity, good biocompatibility and easy surface modification.^13^ Moreover, the bactericidal activity of CQDs is dependent on the chemistry and size of their surface.^14^ For CQDs doped with nitrogen and sulfur, electrostatic interaction mainly contributes to their antibacterial properties and may also give them the ability to activate ROS.^14^ CQDs doped with nitrogen and sulfur show size-dependent growth inhibition of bacteria, which may contribute to the destruction of bacterial membranes *via* high positive charges. Li *et al.* used the pyrolysis of lysine and arginine to synthesize two types of carbon quantum dots, which displayed antibacterial activity against both Gram-negative and Gram-positive bacteria distinctively. They also effectively inhibited the formation of bacterial biofilms without drug resistance.^15^

Nanocomposites can be composited with silver nanoparticles and carbon quantum dots for a more rational design, which combines the biological properties of carbon quantum dots with the antibacterial properties of silver nanoparticles for potential improvement.^16^ The produced material also has a strong bactericidal effect on antibiotic-resistant bacteria. In this work, we synthesized a AgNPs@S,N-CQDs composite through a one-step synthetic process and their antibacterial and cytotoxic properties were systematically investigated. The as-synthesized composite showed excellent antibacterial properties against Gram-negative/Gram-positive bacteria and fungi.

## 2. Experimental section

### 2.1. Synthesis of S,N-CQDs

0.6 g citric acid and 0.195 g β-mercaptoethylamine were dissolved in 10 mL pure water and heated to 150 °C for 3 h in an autoclave. After the reaction, the solution was dialyzed using a 1000 D dialysis bag for 24 h and the dialysate was changed every 6 h to remove unreacted raw materials. Then, the solution was freeze-dried to obtain a solid product.^17^

### 2.2. Synthesis of AgNPs@S,N-CQDs

**Scheme 1:**
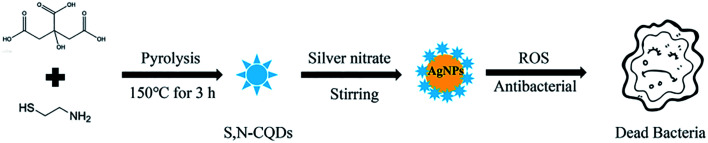
Synthesis of AgNPs@S,N-CQDs active against bacteria.

The silver nitrate solution was added to the S,N-CQD solution under magnetic stirring with a mass ratio of 1 : 1. After stirring for 48 h, the unreacted silver ions were removed by dialysis for 24 h and the dialysate was changed every 6 h to remove unreacted silver nitrate. The resulting solution was freeze-dried to obtain light yellow particles (Scheme 1).^11^

### 2.3. Characterization

The as-synthesized S,N-CQDs and AgNPs@S,N-CQDs were characterized using UV-Visible absorption spectroscopy (Shimadzu, Japan) and fluorescence spectroscopy (Thermo Fisher Scientific, China). The Fourier transform infrared (FTIR) spectra were determined on an IRAffinity-1 spectrophotometer (Shimadzu, Japan). X-ray diffraction (XRD) patterns were measured using a D/max/-2200/PC (Rigaku Corporation, Japan). High-resolution transmission electron microscopy (HRTEM) of AgNPs@S,N-CQDs was performed using a H-7650 (Hitachi, Japan). In addition, X-ray photoelectron spectroscopy (XPS) was performed on an ESCALAB 250 XI (Thermo Fisher Scientific, China). Scanning electronic microscopy (SEM) images and energy-dispersive spectroscopy (EDS) data of AgNPs@S,N-CQDs were obtained using a JSM-7610F PLUS electron microscope (JAPAN). Zeta potential was examined using a Zetasizer Nano ZS90 (Malvern, UK). Raman spectra were recorded using a DXR Raman microscope spectrometer (Thermo Scientific, China).^18^

### 2.4. Antibacterial rate study

Time-kill kinetics studies of S,N-CQDs and AgNPs@S,N-CQDs against different kinds of bacteria were conducted to determine their antibacterial efficiency. Different concentrations of AgNPs@S,N-CQDs or S,N-CQDs (0.032, 0.063, 0.125, 0.25, 0.5, 1, 2, 4 mg mL^−1^) were incubated with different species of bacteria, and the bacterial concentration in the final system was kept at 10^5^ CFU mL^−1^.^19^

### 2.5. Detection of ROS

The steps using the DCFH-DA kit to detect reactive oxygen species involved adding 2 mL of a 10^5^ CFU mL^−1^ MRSA bacterial suspension to an EP tube, then adding AgNPs@S,N-CQDs or S,N-CQDs (0.1 mg mL^−1^). 2 μL DCFH-DA was added to the above solution and the fluorescence intensity was then observed using a fluorescence spectrophotometer with an excitation wavelength of 488 nm.^20^ Rosup was the positive control provided by the DCFH-DA kit. The concentration of AgNPs@S,N-CQDs (5, 25, 50, 100, 200, 400 μg mL^−1^) was changed while the other experimental conditions remained unchanged to detect ROS.

### 2.6. SEM images of the bacteria

SEM was used to observe the surface morphology of the bacteria with or without the AgNPs@S,N-CQDs composite. A 0.3 mL 10^6^ CFU mL^−1^ bacterial suspension was inoculated in 10 mL broth and incubated at 37 °C for 6 h. After that, a 50 μL 1 mg mL^−1^ AgNPs@S,N-CQDs solution was added to 2 mL of the above broth and incubated at 37 °C for 18 h. Then, the broth was centrifuged at 5000 rpm for 5 min to obtain the bacterial precipitate. The bacterial precipitate was mixed in 2.5% aqueous glutaraldehyde at 4 °C to fix for 12 h and dehydrated in alcohol at varying concentrations (50%, 70%, 90% and 100%) for 10 min. The drying bacteria were observed by SEM and the non-treated bacterial suspensions were used as controls.^21^

### 2.7. *In vitro* cytotoxicity test

The *in vitro* cytotoxicity of AgNPs@S,N-CQDs was evaluated by 3-(4,5-dimethylthiazol-2-yl)-2,5-diphenyltetrazolium bromide analysis.^22^ Human hepatoma cells (HepG2 cells) were chosen as the experimental cell type. The experiment was repeated three times. The cell survival rate of the control group was 100%. The following formula was used to calculate the relative cell survival rate (%) after treatment with different concentrations of AgNPs@S,N-CQDs:cell viability (%) = (OD_sample_ − OD_control_)/OD_control_ × 100

## 3. Results and discussion

### 3.1. Characterization of AgNPs@S,N-CQDs

**Fig. 1:**
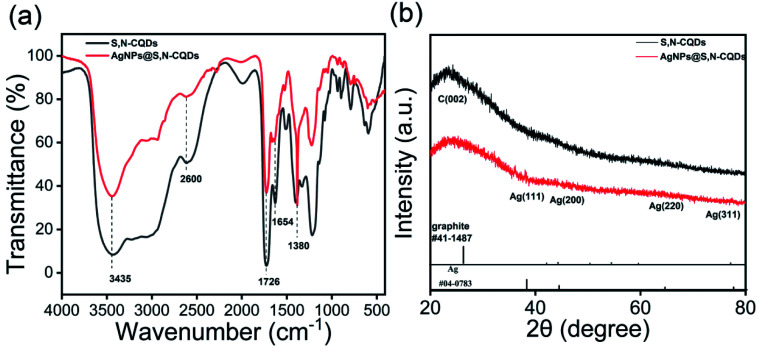
FTIR spectra of S,N-CQDs and AgNPs@S,N-CQDs (a), XRD patterns of S,N-CQDs and AgNPs@S,N-CQDs (b).

In preparing S,N-CQDs, about 0.35 g of sample was obtained after drying 10 mL of the solution. In preparing the AgNPs@S,N-CQDs composite, 0.3 g of the composite was obtained after drying when the masses of silver nitrate and S,N-CQDs were each 0.25 g. When we tested the solubility of the composite, we found that 5 mg of the composite easily dissolved in 1 mL deionized water and no suspended particles were found, indicating that the composite had good water solubility and could be used in the form of a solution for checking bacterial assays. To validate the successful synthesis of S,N-CQDs and the AgNPs@S,N-CQDs composite, the samples were comprehensively characterized using different techniques. S,N-CQDs had a characteristic absorption peak at 350 nm,^23^ which was attributed to the electronic transitions (n–π*) of the C=O groups. Meanwhile, the AgNPs@S,N-CQDs composite material had a characteristic absorption peak at 400 nm, which was the characteristic absorption of silver nanoparticles (Fig. S1A†).^24^ The fluorescence (FL) intensity of the AgNPs@S,N-CQDs (Fig. S1C†) composite was significantly reduced compared with that of S,N-CQDs (Fig. S1B†).^25^ The decrease in the emission intensity can probably be ascribed to the ground state complexation between S,N-CQDs and AgNPs. FTIR spectra (Fig. 1a) were also recorded and the wide absorption band at 3000–3500 cm^−1^ was ascribed to the stretching vibration of OH– and NH–.^26^ The absorption at 1726 cm^−1^ of AgNPs@S,N-CQDs was ascribed to the C=O carbonyl tensile shock, and the decrease in intensity proved that there was an interaction between the oxygen-containing functional groups (–COOH) in S,N-CQDs and AgNPs. The peak at 1380 cm^−1^ was attributed to the stretching vibration of sulfide, and that peak in the AgNPs@S,N-CQDs spectrum may be assigned to the S-related bond,^27^ which also supports the formation of the composite, indicating that the Ag–S bond was generated (Fig. 1b).^11^

**Fig. 2:**
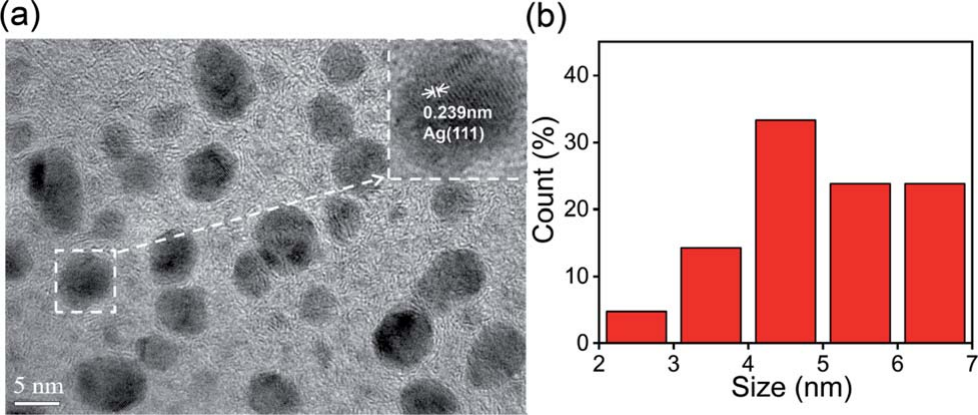
HRTEM image at a scale of 5 nm (a) and particle size distribution (b) of AgNPs@S,N-CQDs.

The XRD patterns of S,N-CQDs and AgNPs@S,N-CQDs (Fig. 1b) correlated well with the standard graphite pattern (41-1487), especially the main characteristic peaks of the (002) plane at 2*θ* = 22° of graphene, which indicated that the prepared S,N-CQDs are graphene-type carbon quantum dots. Crystalline peaks of silver (Ag) were exhibited at 38.12°, 43.98°, 64.66° and 77.62°, corresponding to the (111), (200), (220) and (311) lattice planes, respectively.^11^ The zeta potential (Fig. S2†) of AgNPs@S,N-CQDs was positively charged (18.0 mV), and the value was higher than that of S,N-CQDs (13.7 mV) due to the reduction of AgNPs. The silver content was the highest from the map analysis section (Fig. S4†). The distribution of silver was concentrated in the particles. The AgNPs@S,N-CQDs composite had two peaks at 1360 and 1580 cm^−1^, which were the D peak and G peak of graphene, respectively (Fig. S5†), corresponding to general graphene-based materials.^28^ The AgNPs@S,N-CQDs particles were quasi-spherical, and the particle size was below 10 nm and relatively uniform.^29^ They had a fine fringe pattern with a spacing of 0.239 nm, corresponding to Ag(111) lattice fringes, measured from the HRTEM image (Fig. 2a).^25^ The size distribution histogram (Fig. 2b) reflects the size distribution of AgNPs@S,N-CQDs ranging between 2 nm and 7 nm.^11^

**Fig. 3:**
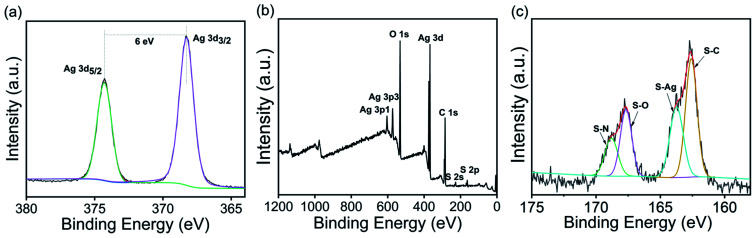
XPS full scan spectrum (a) and deconvoluted high-resolution Ag_3d_ (b) and S_2p_ (c) spectra of AgNPs@S,N-CQDs.

The deconvoluted high-resolution Ag_3d_ spectrum of the AgNPs@S,N-CQDs composite showed typical double peaks at 368.30 and 374.29 eV attributed to Ag 3d_5/2_ and Ag 3d_3/2_, respectively, indicating the successful reduction of Ag^+^ to AgNPs (Fig. 3b).^30^ The binding energy split of the 3d doublet was about 6 eV, indicating that the composite retains the metallic nature of AgNPs.^31^ One peak was found at 163.9 eV in the S_2p_ spectrum (Fig. 3c),^32^ which was ascribed to the binding energy of the Ag–S bond.^11^

### 3.2. Antibacterial activities of AgNPs@S,N-CQDs

According to the MIC value results in Table S1,† when the AgNO_3_ : S,N-CQDs reaction ratio was 1 : 1, the lowest MIC value of 32 μg mL^−1^ was observed. According to the MIC value results in Table S2,† the MIC values of AgNPs@N-CQDs and AgNPs@CQDs were higher than that of AgNPs@S,N-CQDs, indicating that AgNPs@S,N-CQDs had better antibacterial performance compared to the other two groups (Table 1).

**Table 1:** Comparison of previously reported antibacterial nanomaterials and our purposed AgNPs@S,N-CQDs composite^a^

Materials	MIC (μg mL^−1^)				Ref.
	*S. aureus*	*E. coli*	MRSA	*C. albicans*	
Ag/GO	—	440	—	—	33
Ag,N-CQDs	200	250	—	—	34
Ag@CDs	42	42	—	—	35
Ag@S-GQDs	35	70	—	—	11
AgNPs@S,N-CQDs	63	32	63	32	This study

a “—” – not detected.

**Fig. 4:**
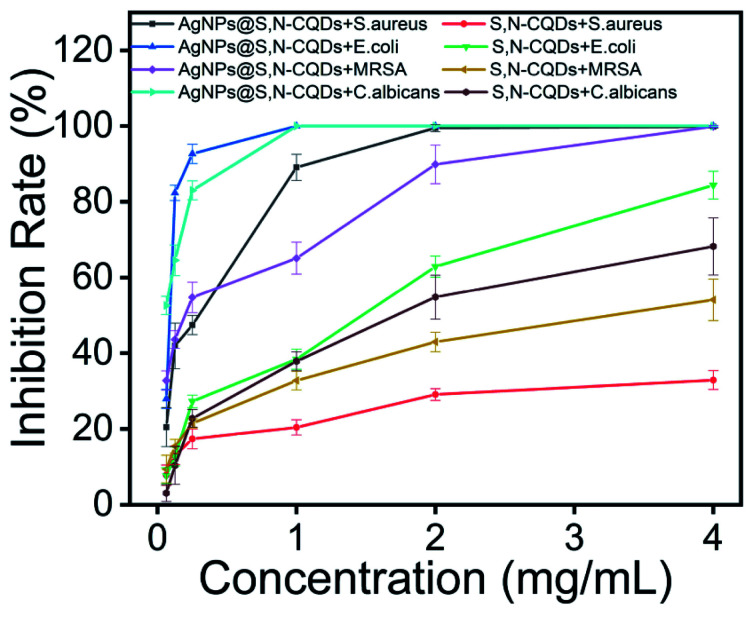
Inhibition rate of different concentrations of AgNPs@S,N-CQDs or S,N-CQDs for *S. aureus*, *E. coli*, MRSA, and *C. albicans*.

The bacteria-inhibiting capability was examined by investigating the inhibition percentages of *S. aureus*, *E. coli*, MRSA, and *C. albicans* incubated in the absence and presence of S,N-CQDs or AgNPs@S,N-CQDs. As can be seen from the antibacterial rate graph of the plate experiment (Fig. S7†), the two groups of samples had certain antibacterial ability against Gram-positive bacteria, Gram-negative bacteria, fungi, and antibiotic-resistant bacteria. The inhibition rates for *S. aureus*, *E. coli*, and *C. albicans* almost approached 100% and the rate for MRSA was about 85% when the concentration of AgNPs@S,N-CQDs was 2 mg mL^−1^, while the inhibition rate of S,N-CQDs was below 60%. Thus, the high-efficiency antibacterial material AgNPs@S,N-CQDs has better antibacterial properties than S,N-CQDs (Fig. 4).

### 3.3. Antibacterial mechanism of AgNPs@S,N-CQDs

#### 3.3.1. Detection of ROS

**Fig. 5:**
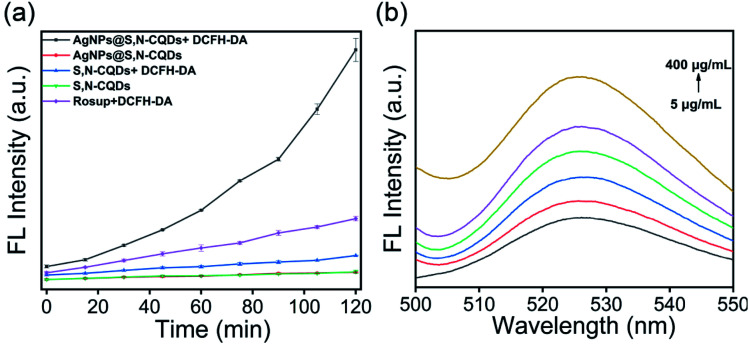
Fluorescence of DCF incubated with S,N-CQDs or AgNPs@S,N-CQDs (a), fluorescence of DCF incubated with different concentrations of AgNPs@S,N-CQDs (b). The amount of ROS was proportional to the fluorescent intensity of DCF (the concentrations were 5, 25, 50, 100, 200, and 400 μg mL^−1^).

To determine the ROS generation, the DCFH-DA probe was adopted. Compared to the Rosup group (positive control), the FL intensity had a faster increase in the presence of AgNPs@S,N-CQDs, indicating that the ROS increased. The S,N-CQDs also produced a small amount of ROS, indicating that AgNPs@S,N-CQDs was conducive to the generation of ROS. The experimental results showed that the antibacterial mechanism of AgNPs@S,N-CQDs may be due to the generation of ROS. Different concentrations of AgNPs@S,N-CQDs were incubated with DCFH-DA for 90 minutes to detect the change in fluorescence. With increasing concentration, the fluorescence gradually increased, indicating that the generation of ROS was related to the concentration of AgNPs@S,N-CQDs (Fig. 5b).

#### 3.3.2. Detection of ˙OH

**Fig. 6:**
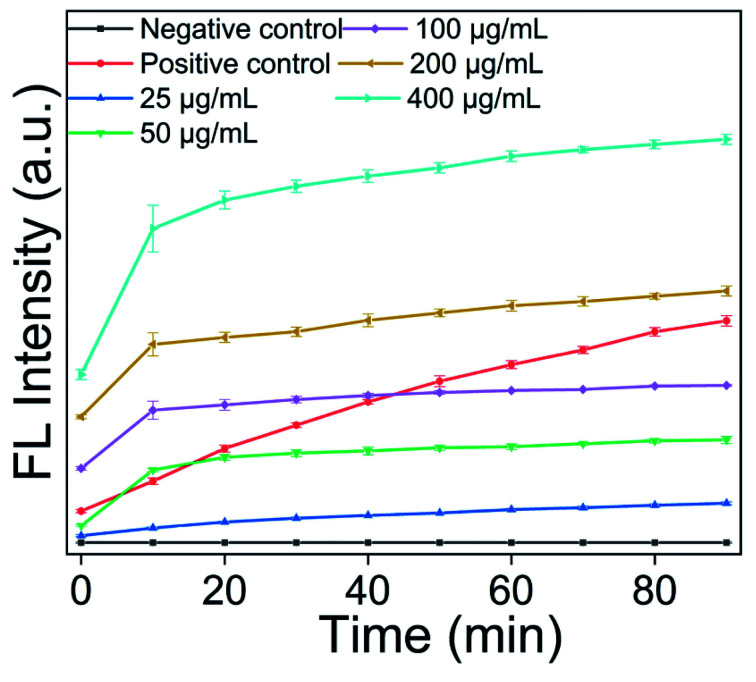
Fluorescence curve of CCA after adding different concentrations of AgNPs@S,N-CQDs.

To further explore the mechanism of the antibacterial ability of AgNPs@S,N-CQDs, we investigated the types of ROS generated, which were found to be hydrogen peroxide (H_2_O_2_) and hydroxyl radicals (˙OH). Hydroxyl radicals can oxidize coumarin-3-carboxylic acid (CCA) to 7-hydroxy-coumarin-3-carboxylic acid (7-OH-CCA), which is fluorescent. The positive control was a mixed solution of divalent iron ions and hydrogen peroxide based on the Fenton reaction to generate hydroxyl radicals. When a solution of AgNPs@S,N-CQDs was added to the CCA solution, growing fluorescence intensity was observed (Fig. 6). With the passage of time or increase in the concentration of AgNPs@S,N-CQDs, the growth rate of the fluorescence intensity also increased. Horseradish peroxidase catalyzes the oxidation of 4-hydroxyphenylacetic acid (HPA) by H_2_O_2_ to produce a fluorescent dimer (*λ*_em_ = 450 nm), which can be used as a detection reagent to determine the production of hydrogen peroxide in the solution. In the presence of AgNPs@S,N-CQDs (25 μg mL^−1^, Fig. S6†), fluorescence was observed, indicating that AgNPs@S,N-CQDs promoted the production of H_2_O_2_.^20^

### 3.4. Bactericidal behavior of AgNPs@S,N-CQDs

**Fig. 7:**
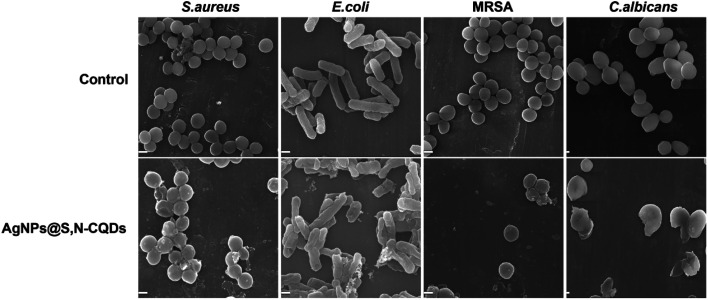
SEM images of *S. aureus*, *E. coli*, MRSA and *C. albicans* before (control) and after treating with AgNPs@S,N-CQDs (100 μg mL^−1^). Scale bars: 1 μm.

As shown in the SEM images (Fig. 7), the bacteria in the control group retained a complete, compact and typical shape. In contrast, there were distinct wrinkles and ruptures in the bacteria when exposed to AgNPs@S,N-CQDs. At the same time, a great amount of AgNPs@S,N-CQDs was observed on the bacterial surface. Taking *S. aureus* and *E. coli* as examples, the zeta potential of the bacterial membrane surfaces was negative. After treating with AgNPs@S,N-CQDs, the absolute zeta potential of the bacteria was lower than before (Fig. S8†), which was attributed to the strong adhesion action between the negatively charged membrane surfaces of bacterial cells and AgNPs@S,N-CQDs *via* electrostatic interactions.

### 3.5. Cytotoxicity assay

**Fig. 8:**
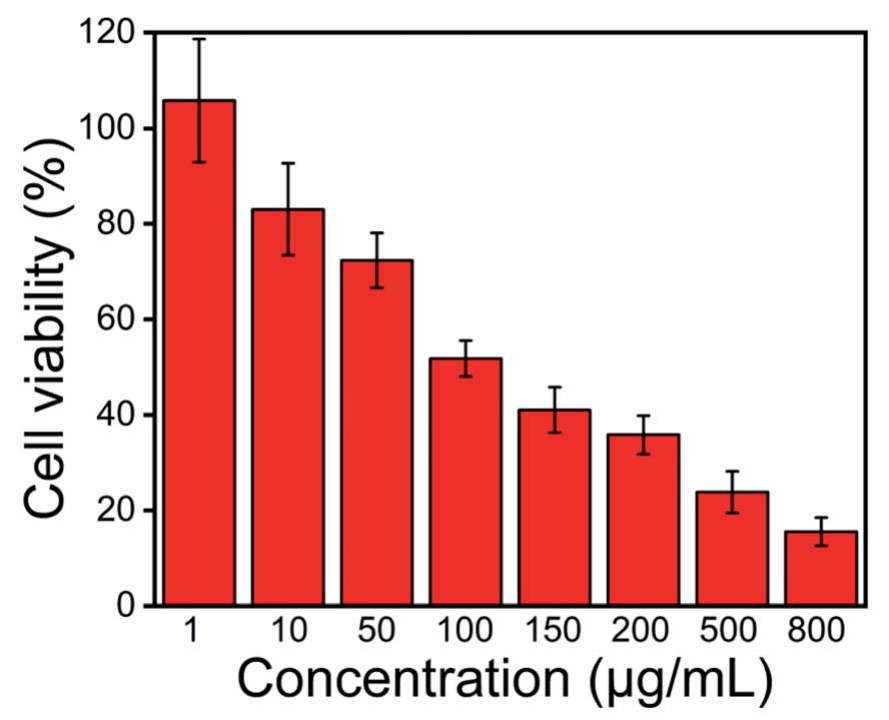
Relative viability of HepG2 cells treated with different concentration of AgNPs@S,N-CQDs for 24 h.

Biocompatibility is an important property in evaluating the potential biomedical application of new materials. The biocompatibility assay of AgNPs@S,N-CQDs was performed on HepG2 cells and is shown in Fig. 8. Different concentrations (1, 10, 50, 100, 150, 200, 500 and 800 μg mL^−1^) of AgNPs@S,N-CQDs were added in a 96-well plate containing HepG2 cells (5 × 10^3^ cells) and the effect was observed after 24 h. It was found that the viability of cells reduced by about 20% in response to the MIC concentration, which showed that the composite still maintained good biocompatibility while exhibiting antibacterial properties. Within the effective antibacterial concentration range, it still maintained high cell viability. As the concentration of the composite material increased, the viability of the cells also decreased gradually. AgNPs@S,N-CQDs might be a promising antibacterial agent with low toxicity. Such cytotoxicity along with antibacterial activity suggests that the AgNPs@S,N-CQDs composite can be an effective next-generation antibacterial agent.

## 4. Conclusion

In summary, a new type of AgNPs@S,N-CQDs composite consisting of AgNPs uniformly decorated by S,N-CQDs and composited with citric acid and mercaptoethylamine, with superior antibacterial properties, was successfully synthesized. The characterization results demonstrated that the AgNPs in the composite were evenly coated by S,N-CQDs. The composite material exhibited good hydrophilicity and broad-spectrum antibacterial properties. The MIC values were 32 μg mL^−1^ for *E. coli* and *C. albicans* and 63 μg mL^−1^ for *S. aureus* and MRSA. The high-efficiency antibacterial activity was detected using the DCFH-DA kit and was found to be due to the production of ROS. The high antibacterial performance came from two types of reactive oxygen species, hydroxyl radicals and hydrogen peroxide, verified through a series of experiments. Additionally, the AgNPs@S,N-CQDs composite revealed superior cell viability in HepG2 cells. The results indicated that the AgNPs@S,N-CQDs composite can be used as a next-generation bio-antibacterial agent for various biomedical applications, including wound dressings, antibacterial coatings, and daily disinfectant chemicals.

## Conflicts of interest

There are no conflicts of interest to declare.

## Supplementary Material

RA-012-D2RA00561A-s001
