# Supplementary material for: Silver nanoparticle@carbon quantum dot composite as an antibacterial agent
Source: RSC Adv. 2022 Mar 28;12(16):9621–7. doi: 10.1039/d2ra00561a (PMC8959443; doi:10.1039/d2ra00561a)
Supplement: RA-012-D2RA00561A-s001 [file RA-012-D2RA00561A-s001.pdf]

Electronic Supplementary Material (ESI) for RSC Advances.
This journal is © The Royal Society of Chemistry 2022

Supporting Information

Silver Nanoparticles@carbon quantum Dots Composite as Antibacterial Agent

Tianyu Liu[a], Qianyue Pang[b], Kang Mai[c], Xiaoting He[b], Li Xu*[a b], Feiyan Zhou*[d], Yi Liu*[a b]

[a] *School of Pharmacy, Guangdong Pharmaceutical University, Guangzhou 510006, China*

[b] *School of Pharmaceutical and Chemical Engineering, Guangdong Pharmaceutical University, Zhongshan 528458, China*

[c] *Zhongshan Carefor Daily Necessities Ltd, Zhongshan 528000, China*

[d] *Guangzhou Baiyunshan Weiyi Industrial Co., Ltd, Guangzhou 510000, China*

EXPERIMENTAL SECTION

1. Material and regents

Citric acid (CA, 99.5%) and β-mercaptoethylamine (97%) were purchased from Rhawn Reagent Company. $AgNO_3$(97-98%) came from Shanghai insitute of fine chemicals. The test bacteria, Staphylococcus aureus (*S. aureus*, ATCC25923) and Escherichia coli (*E. coli*, ATCC25922), Methicillin-resistant Staphylococcus aureus (MRSA, ATCC33591), Candida albicans *(C.albicans*, ATCC10231)were obtained from Guangdong Microbial Culture Collection Center. LB broth, potato dextrose agar, nutrient agar, sabouraud dextrose broth were purchased from Guangdong Huankai microbial Sci. Tech. Co. Ltd. All reagents were of analytical grade and no processing

was done before using. Reactive oxygen species (ROS) detection kit was purchased from Beyotime Biotechnology Company.

2. Synthesis of CQDs

The β-mercaptoethylamine was replaced by ethylenediamine, and the rest of the conditions were unchanged to prepare N-CQDs. The β-mercaptoethylamine was removed, and the rest of the preparation conditions remain unchanged to prepare CQDs. CQDs and N-CQDs were respectively reacted with silver nitrate in a mass ratio of 1:1, and other reaction conditions remained unchanged to prepare AgNPs@N-CQDs and AgNPs@CQDs.

3. The test of minimum inhibitory concentration (MIC)

Before the MIC experiment, the bacteria on the solid medium, including *S. aureus*, MRSA, and *E. coli*, were cultured in liquid LB broth for 18 h. *C.albicans* cultured in Sabouraud glucose liquid medium for 24h. In a sterile 96-well plate, 100 μL of LB broth were added to each well and added a certain concentration of S,N-CQDs or AgNPs@S,N-CQDs composite to the first well. A serial two-fold dilutions were made, then adding 100 μL of the bacterial suspension with a concentration of 2×10^5 CFU/mL to wells. MIC was measured by turbidity method, that is, the concentration of S,N-CQDs or AgNPs@S,N-CQDs corresponding to the well without turbidity was the MIC value. All experiments were performed in triplicate.[1]

4. Detection of ·OH

Coumarin-3-carboxylic acid (CCA) is a chemical probe that can react with hydroxyl radicals to generate fluorescent 7-hydroxycoumarin 3-carboxylic acid (7-OH-CCA),

which is used to determine whether AgNPs@S,N-CQDs can produce ·OH or not. 100 μM CCA, 10 mM sodium hydroxide and AgNPs@S,N-CQDs of different concentrations were contained in 2 mLH_2O. The fluorescence was detected at 10 minutes intervals and the fluorescence spectra were recorded (λex = 340 nm, λem = 450 nm).[2]

5. Detection of H_2O_2

A horseradish peroxidase (HRP)-based fluorescence assay was used to examine if the AgNPs@S,N-CQDs generated H_2O_2. 2 mL 0.1 M Tris buffer (pH 8.8) which was contained 1 mM 4-hydroxyphenylacetic acid (HPA), 0.04 mg/mL HRP and different concentrations of AgNPs@S,N-CQDs were tested every 10 min. Then its fluorescence spectrum was recorded (λex = 316 nm, λem = 450 nm).[2]

6. In vitro cytotoxicitytest

First, HepG2 cells (5×10^3 cells per well) were seeded in a 96-well plate and cultured for 24 h. The culture conditions were in Dulbecco's Modified Eagle Medium (DMEM), which contained 10% fetal bovine serum (FBS) and 1% penicillin-streptomycin. The cells were incubated in 37°C incubator with 5% CO_2supply. Next, AgNPs@S,N-CQDs solutions with different concentrations were placed in a 96-well plate. And the untreated group was used as the control group. The samples were then incubated in a 5% CO_2 incubator for 24 h, then the culture medium was discarded.70 μL DMEM and 30 μL 0.5% MTT were added to each well. Soon afterwards, the cells were cultured in the incubator for 4 h. After that, the MTT solution in each well was

discarded and200 μL dimethyl sulfoxide (DMSO) was addedto detect the absorbanceat 570 nm by a microplate reader.[3]

Table S1 The MIC value of different groups of composite materials prepared with different raw material reaction mass ratios (mg/mL)

	$AgNO_3$:S,N-CQDs				
	1:4	1:2	1:1	2:1	4:1
S. aureus	1	0.5	0.063	0.063	0.063
MRSA	1	0.5	0.063	0.063	0.063
E.coli	1	0.5	0.032	0.063	0.063
C.albicans	1	0.5	0.032	0.063	0.063

Table S2 The MIC value of different kinds of composite materials prepared by using different CQDs (mg/mL)

	AgNPs@CQDs	AgNPs@N-CQDs	AgNPs@S,N-CQDs
S. aureus	2	2	0.063
MRSA	2	2	0.063
E.coli	2	2	0.032
C.albicans	2	2	0.032

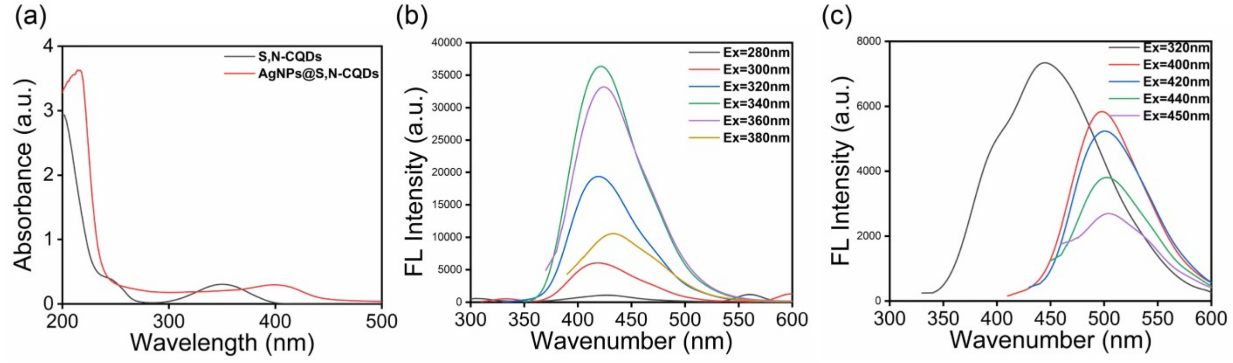

Figure S1 UV-Vis absorption spectra (a) of S,N-CQDs (1 mg/mL) and AgNPs@S,N-CQDs (1 mg/mL) and fluorescence spectrum of S,N-CQDs (0.1 mg/mL, b) and AgNPs@S,N-CQDs (0.1 mg/mL, c)

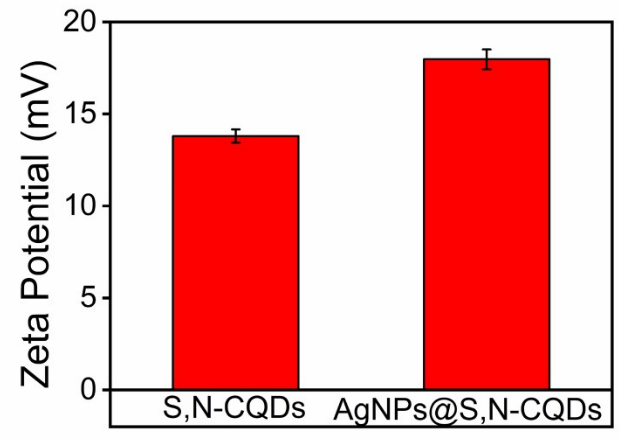

Figure S2 Zeta potential of S,N-CQDs (1 mg/mL) and AgNPs@S,N-CQDs (1 mg/mL)

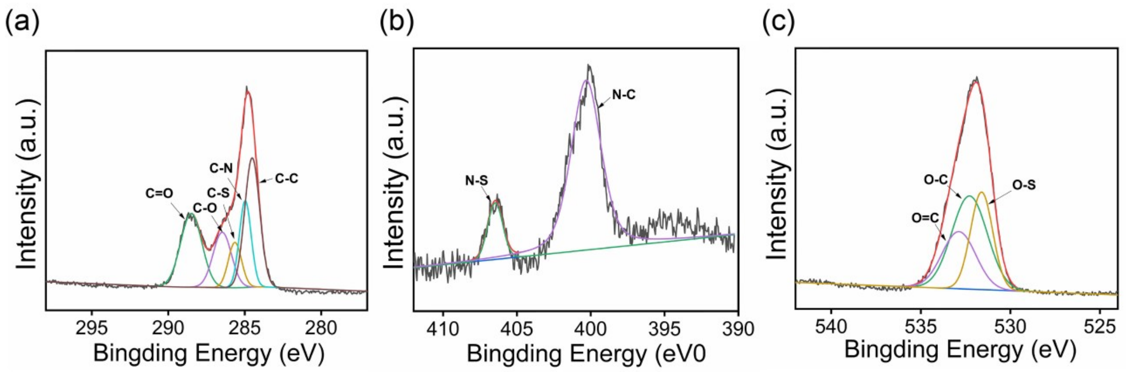

Figure S3 Deconvoluted high-resolution of C_{1s}(a), N_{1s}(b), O_{2p}(c) spectra of AgNPs@S,N-CQDs

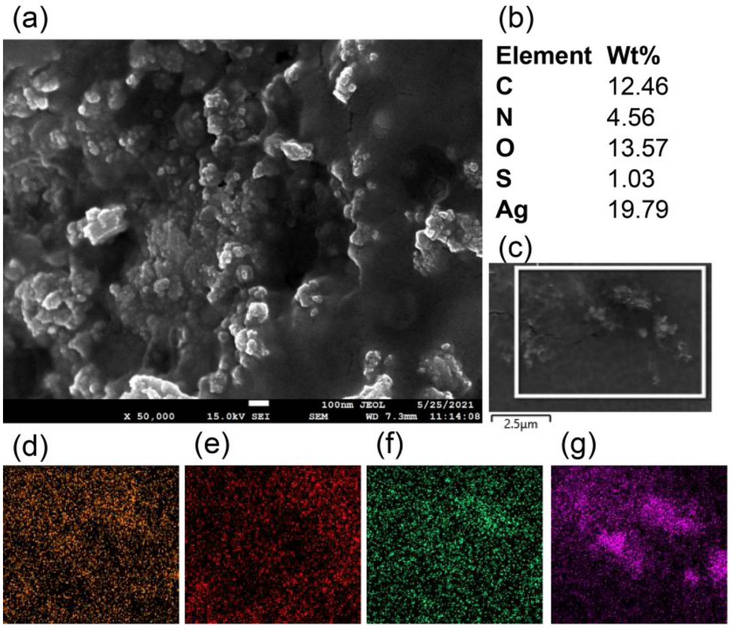

Figure S4 SEM image of AgNPs@S,N-CQDs composite material (a), Element content table (b), map analysis section (c), C element distribution (d), O element distribution (e), S element distribution (f), Ag element distribution (g)

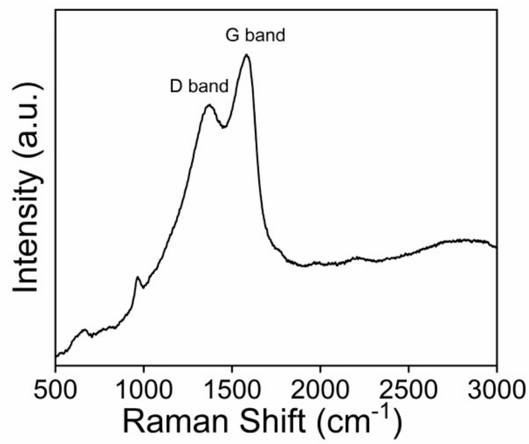

Figure S5 Raman curve of AgNPs@S,N-CQDs

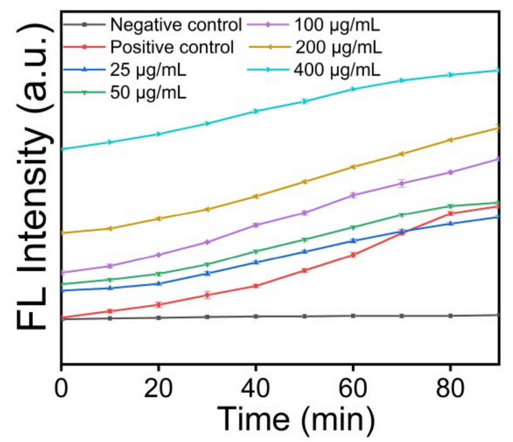

Figure S6 Fluorescence curve offluorescent intensity after adding different concentrations of AgNPs@S,N-CQDs

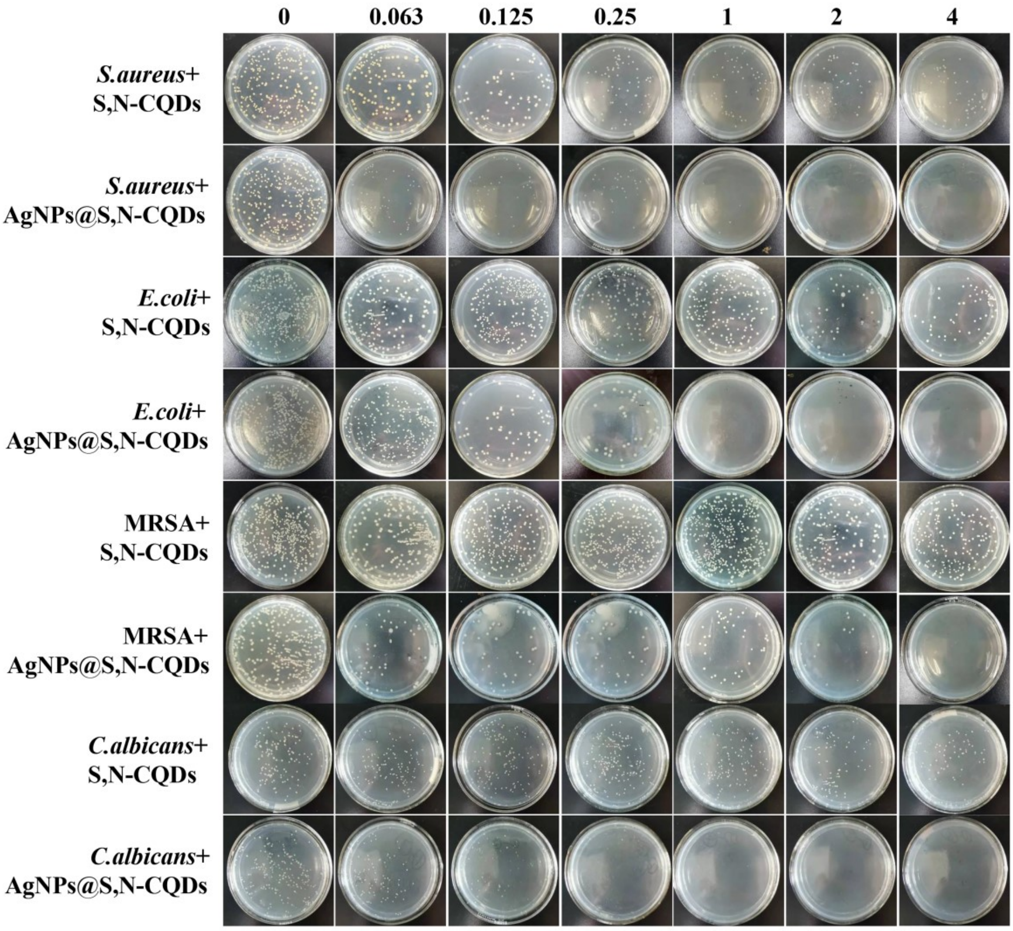

Figure S7 Images of bacterial suspension of *S.aurus*, *E.coli*, MRSA or *C.albicans* before and after treatment with different concentration S,N-CQDs or AgNPs@S,N-CQDs.(0, 0.063, 0.125, 0.25, 1, 2, 4 mg/mL)

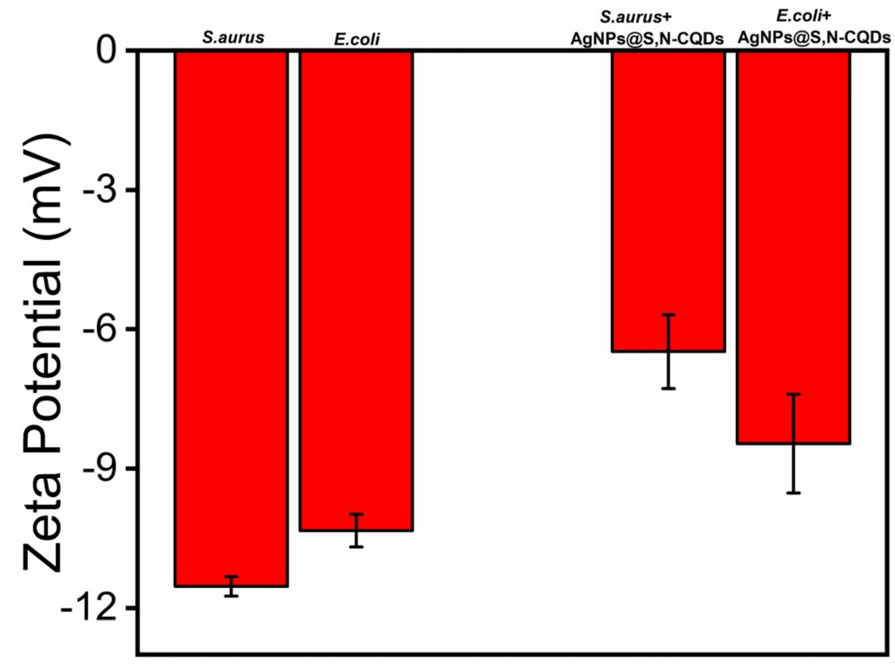

Figure S8 Zeta potential of *S.aurus*, *E.coli* before and after cultivated with AgNPs@S,N-CQDs (0.1 mg/mL) for 2 h

References

1. P. Li, S. Liu, W. Cao, G. Zhang, X. Yang, X. Gong and X. Xing, *Chem. Commun. (Cambridge, U. K.)*, 2020, **56**, 2316-2319.
2. X. Nie, C. Jiang, S. Wu, W. Chen, P. Lv, Q. Wang, J. Liu, C. Narh, X. Cao, R. A. Ghiladi and Q. Wei, *J. Photochem. Photobiol., B*, 2020, **206**, 111864.
3. S. Kadian, G. Manik, N. Das, P. Nehra, R. P. Chauhan and P. Roy, *J. Mater. Chem. B*, 2020, **8**, 3028-3037.
